# CRISPR-Based Genome Editing for Nutrient Enrichment in Crops: A Promising Approach Toward Global Food Security

**DOI:** 10.3389/fgene.2022.932859

**Published:** 2022-07-14

**Authors:** Dileep Kumar, Anurag Yadav, Rumana Ahmad, Upendra Nath Dwivedi, Kusum Yadav

**Affiliations:** ^1^ Department of Biochemistry, University of Lucknow, Lucknow, India; ^2^ Department of Microbiology, College of Basic Science and Humanities, Sardarkrushinagar Dantiwada Agriculture University, Banaskantha, India; ^3^ Department of Biochemistry, Era Medical University and Hospital, Lucknow, India

**Keywords:** biofortification, biofortified crops, CRISPR-cas system, genome editing, hidden hunger, malnutrition, micronutrients

## Abstract

The global malnutrition burden imparts long-term developmental, economic, social, and medical consequences to individuals, communities, and countries. The current developments in biotechnology have infused biofortification in several food crops to fight malnutrition. However, these methods are not sustainable and suffer from several limitations, which are being solved by the CRISPR-Cas-based system of genome editing. The pin-pointed approach of CRISPR-based genome editing has made it a top-notch method due to targeted gene editing, thus making it free from ethical issues faced by transgenic crops. The CRISPR-Cas genome-editing tool has been extensively used in crop improvement programs due to its more straightforward design, low methodology cost, high efficiency, good reproducibility, and quick cycle. The system is now being utilized in the biofortification of cereal crops such as rice, wheat, barley, and maize, including vegetable crops such as potato and tomato. The CRISPR-Cas-based crop genome editing has been utilized in imparting/producing qualitative enhancement in aroma, shelf life, sweetness, and quantitative improvement in starch, protein, gamma-aminobutyric acid (GABA), oleic acid, anthocyanin, phytic acid, gluten, and steroidal glycoalkaloid contents. Some varieties have even been modified to become disease and stress-resistant. Thus, the present review critically discusses CRISPR-Cas genome editing-based biofortification of crops for imparting nutraceutical properties.

## Introduction

Malnutrition is becoming a rapidly increasing and serious problem as the world’s population grows. The world’s population will reach 8.3 billion in 2030, up from 7.8 billion at present. According to estimates, almost 800 million people are malnourished worldwide, with 98 percent living in underdeveloped countries (Sinha et al., 2019). Undernutrition (wasting, stunting, and being underweight), insufficient vitamins and minerals, obesity, and the consequent diet-related non-communicable disorders are all examples of malnutrition. Furthermore, more than 340 million people suffer from one or more micronutrient deficiencies, including deficiencies in vitamin A, iron, iodine, and zinc (UNICEF, 2021).

Fortification and biofortification are food enrichment technologies that differ in their approach. In the former method, fortificants are added directly to the food during processing, but the latter involves fortification at the crop production level. In comparison to fortification, biofortification is cost-effective as it is a one-time investment to develop a biofortified crop and recurrent costs are low (Shoab and Hefferon, 2022). Biofortified crops hold a brighter future to address nutritional challenges (Yadav et al., 2020; Buturi et al., 2021; Mushtaq and Nazir, 2021; Ziarati et al., 2021). Biofortification is considered a sustainable and long-term solution to provide micronutrient-rich crops to people (Bouis and Welch, 2010; Bouis and Saltzman, 2017; Garg et al., 2018; Heck et al., 2020; Van Der Straeten et al., 2020).

The crops are biofortified for desired nutrients through nutrient treatments as well as breeding (Garg et al., 2018). Agronomic biofortification involves the deliberate use of mineral fertilizers to increase the concentration of a target mineral in edible portions of crops (Adu et al., 2018). Advanced agronomic biofortification includes engineered nanoparticles attached with fertilizers (nano fertilizers) and PGPR (plant growth–promoting rhizobacteria) (Nayana et al., 2020). Moreover, crucial quantitative trait loci (QTLs) are also utilized in crop breeding programs to improve crop nutrient profiles (Gangashetty et al., 2016). Nevertheless, plant breeding, especially polyploid crop breeding, is a time-consuming and laborious method for improving crop productivity (Parry et al., 2009; Nagamine and Ezura, 2022). Some crops have also been biofortified for desired nutrients through transgenic technology-based genetic alterations (Pérez-Massot et al., 2013). Disadvantages of genetically modified (GM) crops include allergic reactions in humans and reduced nutrition. Also, they cause environmental impact by releasing toxins in the soil, induce pest resistance, and disruption of crop biodiversity. Several ethical concerns are associated with GM crops.

In view of the disadvantages of GM crops, genome editing (GE) technology offers distinct advantages (Gaj et al., 2013; Xiong et al., 2015; Aglawe et al., 2018; Fiaz et al., 2021; Nagamine and Ezura, 2022). Thus, genome editing produces predictable and inheritable mutations in specified regions of the genome, with minimal off-target effects and no external gene sequence integration (Bhattacharya et al., 2021). Deletions, insertions, single-nucleotide substitutions, and extensive fragment substitutions are used for GE-mediated DNA alterations. Systems such as homing endonucleases or meganucleases (HEs) (Daboussi et al., 2015), Zinc-Finger Nucleases (ZFNs) (Urnov et al., 2010), and transcription activator-like effector nucleases (TALENs) (Joung and Sander, 2013) were engaged as genome editing tools before the discovery of CRISPR-associated protein (Cas) (Wang et al., 2016). The brief details of previously used genome editing methods are described below.

Meganucleases, also known as homing endonucleases, are rare-cutting enzymes found in all microbial genomes. These enzymes identify and cleave lengthy DNA sequences (usually 18–30 base pairs), resulting in double-strand DNA breaks (DSBs). Various designed meganuclease variants are available to cleave unique DNA targets for genomic changes for creating important characteristics in crop species (Daboussi et al., 2015). Homing endonucleases technology has suffered from technical problems in the manufacture of these nucleases and designing vectors for their entrance into cells and off-targeting consequences (Jin et al., 2016; Rey-Rico and Cucchiarini, 2016).

Zinc-finger nucleases (ZFNs) are “nucleases” consisting of engineered zinc-finger DNA-binding domains paired with a nuclease, most often the FokI nuclease. ZFN-induced double-strand breaks are exposed to cellular DNA repair processes, resulting in remarkably targeted mutagenesis and targeted gene replacement (Carroll, 2011). The zinc-finger domains consist of four to six 30 amino acid domains that may bind to trinucleotide sequences, limiting total DNA-binding domain specificity to 12–18 nucleotide sequences (Davies et al., 2017). However, ZFN technology has disadvantages such as complex design (which requires customized protein for each DNA sequence), low engineering feasibility, low specificity, normal efficacy, and inability to gene knockout and RNA editing (Zhang J.-H. et al., 2016; Zhang B. et al., 2016; Callaway, 2016; Wang et al., 2016; Salsman and Dellaire, 2017; Sun et al., 2018).

Transcription activator-like effector nucleases (TALENs) are a nonspecific DNA-cleaving nuclease linked to a DNA-binding domain that could be tailored to target specific sequences. TALENs are made up of an engineered array of TALE repeats fused to the FokI nuclease domain and are used for editing the genome (Reyon et al., 2013). However, TALENs also have disadvantages such as complex design, lower engineering feasibility, lower specificity, low efficacy, and inability to gene knockout and RNA editing (Zhang J.-H. et al., 2016; Zhang B. et al., 2016; Callaway, 2016; Wang et al., 2016; Salsman and Dellaire, 2017; Sun et al., 2018).

Thus, CRISPR-Cas technology has emerged as a promising genome editing tool overcoming many of the abovementioned disadvantages of HEs, ZFNs, and TALENs. Therefore, it is currently the most extensively used genome editing technique worldwide because of its simple design, cost-effectiveness, high efficiency, good reproducibility, high engineering feasibility, ability to create gene knockout, RNA editing, and quick cycle (Asmamaw and Zawdie, 2021).

Thus, the present review critically discusses CRISPR-Cas genome editing-based biofortification of crops with respect to enhancement of carbohydrate, protein, fatty acids, secondary metabolite contents, as well as imparting disease and stress-resistance.

## Overview of the CRISPR-Cas Systems

Most bacteria and Archaea have an adaptive defense system called CRISPR-Cas that protects them from phages, viruses, and other foreign genetic material (Marraffini and Sontheimer, 2010). The main components of the CRISPR-Cas9 system are an RNA-guided Cas9 endonuclease and a single-guide RNA (sgRNA). Type II CRISPR-Cas9 is one of the best-defined and most commonly utilized categories in multiple CRISPR-Cas systems (Jiang and Doudna, 2017). Cas9 endonucleases HNH domain cuts one strand of sgRNA, while the RuvC-like domain cuts the opposite strand of dsDNA, resulting in double-strand breaks (DSBs). As a result, the plant endogenous repair system automatically repairs DSBs *in vivo*, utilizing error-prone non-homologous end-joining (NHEJ) or homology-directed repair (HDR), resulting in massive insertion or fragment replacement (Liu X. et al., 2017).

**FIGURE 1:**
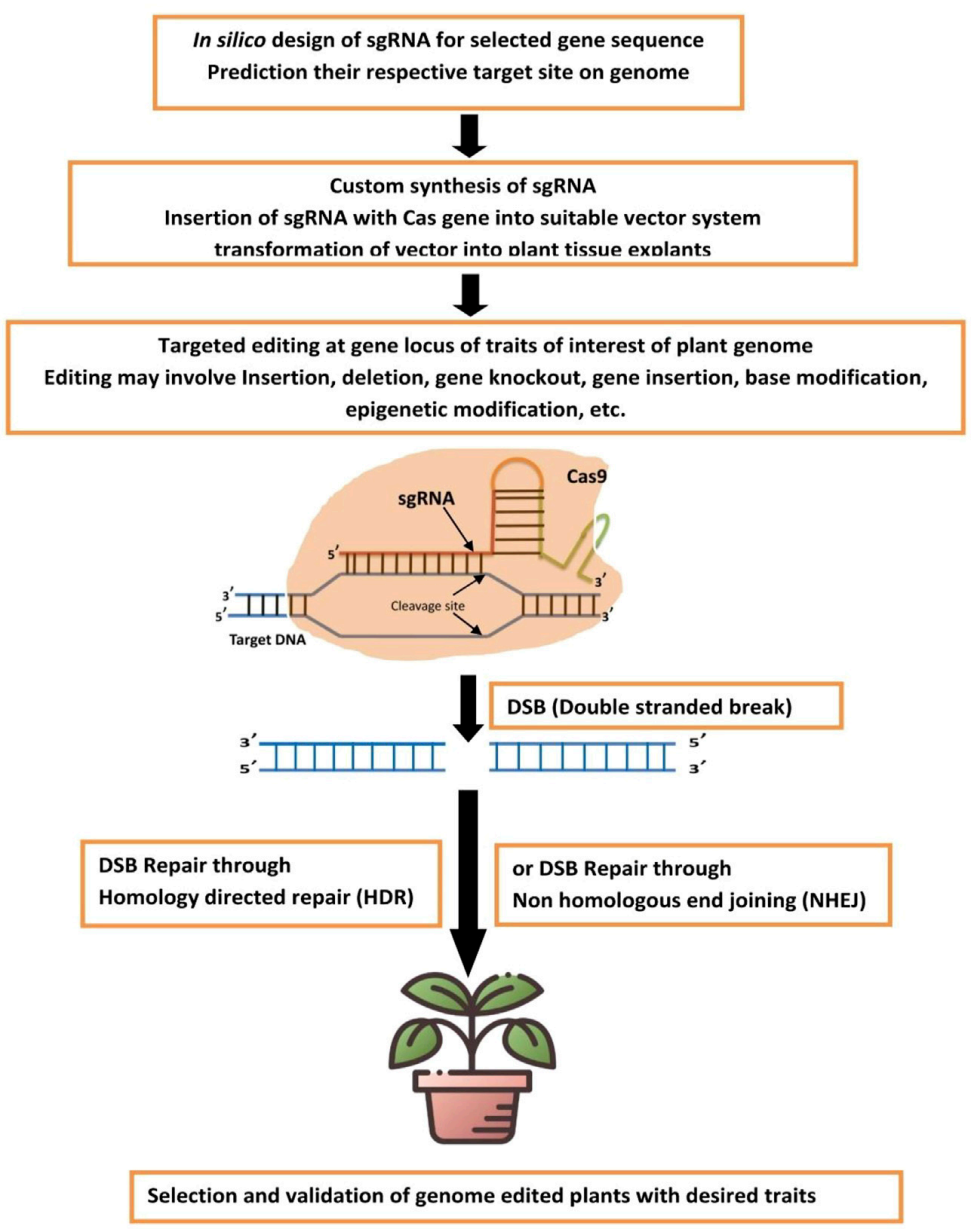
Schematic representation for CRISPR-Cas genome editing methodology for developing genetically edited biofortified plants.

The schematic representation of the CRISPR-Cas genome editing methodology for developing genetically edited biofortified plants is shown in Figure 1.

Efforts have been made by researchers to modify the CRISPR-Cas system to make its application more efficient and specific as depicted in Table 1.

**TABLE 1:** Modifications in the CRISPR-Cas system.

CRISPR-Cas system	Modification	Effect	References
Cas9^D10A^ nickase	By point mutations D10A of RuvC domain of Cas9	For minimizing off-target effects and cleaving the single strand	Ran et al. (2013)
Dead Cas9 (dCas9)	By two-point mutations H841A and D10A, into HNH and RuvC nuclease domain of Cas9, respectively	For transcriptional activation, inhibition, and epigenetic modification	Qi et al. (2013), Dominguez et al. (2016)
	CRISPRa-fusing transcriptional activators and dCas9		
	CRISPRi-fusing transcriptional repressors and dCas9		
Base editing system	Cytidine base editor (CBE) developed by fusion of APOBEC1 to dCas9	For site-directed mutagenesis as CBE causes C_G to T_A point mutations, ABE causes A_T to G_C, and CRISPR-BETS causes stop codon	Komor et al. (2016), Gaudelli et al. (2017), Wu et al. (2021)
	Adenine base editor (ABE) developed by fusion of TadA to dCas9		
	CRISPR-BETS (base editing to stop)		
Cas 9 variants	xCas9 developed by the phage-assisted continuous evolution (PACE) system, SpCas9-NG developed the PACE-system such as SpCas9-NRRH, SpCas9-NRCH, SpCas9-NRTH, SpG, SpRY, etc.	xCas9 recognizes the multiple PAMs other than NGG-PAM (NG, GAA, GAT, etc.), SpCas9-NG recognizes relaxed NG PAMs and SpG recognize near-PAM less and SpRY, recognizes all PAMs (NRN and NYN PAMs)	Hu et al. (2018), Nishimasu et al. (2018), Miller et al. (2020), Walton et al. (2020)
Prime editing system	Composed of pegRNA and reverse transcriptase fused to the C terminus of Cas9 (H841A) nickase	For targeted insertions, deletions, and base-to-base conversions without using donor DNA templates or double-strand breaks (DSBs)	Scholefield and Harrison (2021)
CRISPR tissue–specific knockout (CRISPR-TSKO)	CRISPR-TSKO is generated by expression of Cas9 only in particular tissues using specific promoters	For discovery and analysis of gene functions in specific tissues	Decaestecker et al. (2019)
RNA editing system	CRISPR-Cas13 binds to target single-stranded RNA (ssRNA) and cleaves the target.	For RNA knockdown, transcript labeling, splicing regulation, and virus detection	Abudayyeh et al. (2017), Tang et al. (2021)
	REPAIR RNA base editor (fusion of dcas13 to adenosine deaminase)		
	RESCUE RNA base editor (fusion of dCas13 to cytidine deaminase		

## Development of Biofortified Crops Through CRISPR-Cas Genome Editing Approach

The CRISPR-Cas system allows rapid, site-specific genome modification in a single cell or a whole organism. It regulates transcription, changes epigenomes, runs genome-wide screens, and imaging chromosomes. The CRISPR-Cas system is now increasingly used for developing edited crops due to its diverse applications in genome editing. Using the CRISPR-Cas, research groups have engineered several crop systems for disease resistance (Schenke and Cai, 2020), drought, salinity, and thermotolerance (Chennakesavulu et al., 2021). CRISPR-Cas is aiding in developing climate-ready crops (Razzaq et al., 2021) and improving crop quality parameters such as appearance, palatability, nutritional components, and other preferred traits (Liu Q et al., 2021). This study has reviewed the nutritional enrichment of important crops using the CRISPR-Cas genome editing method.

### Vitamin A Enriched Crops

Carotenoids are widely distributed isoprenoid pigments essential for photosynthetic organisms. Humans do not produce carotenoids *de novo*, but they require them in their food, notably as β-carotene and vitamin A precursors (Maoka, 2020). Vitamin A is necessary for biological functions that include light transduction in vision, embryonic development, immunological operation, and overall health maintenance in human beings (Timoneda et al., 2018). Vitamin A deficiency (VAD) is one of the most severe worldwide health issues, resulting in a variety of symptoms such as xerophthalmia, night blindness, pediatric blindness, and an increased risk of morbidity and mortality, particularly in young children (Sommer, 2008; Reddy et al., 2022). Given this, carotenoid-enriched staple crops (golden crops), either through traditional breeding or genetic engineering, were initiated to combat VAD (Zheng et al., 2020). Among all the genetic engineering techniques, the CRISPR-based genome-editing technique seems the most efficient and widespread method, making rapid, DNA/transgene-free, and targeted multiplex genetic modification of organisms: a reality for developing “golden” staple crops (Zheng X. et al., 2021). Through CRISPR-Cas-based systems, various genome-edited golden crops, that is, carotenoid biofortified crops have been produced to combat VAD (Table 2). For instance, the Golden rice cultivar Kitaake has been developed by Knock-in, a 5.2-kb carotenogenesis cassette consisting of *CrtI* and maize *PSY* genes. The variety contains 7.9 μg/g dry weight (DW) β-carotene in the endosperm (Dong et al., 2020).

**TABLE 2:** Vitamin A enriched biofortified crops *via* CRISPR-Cas systems.

Vitamin A enriched biofortified crops	CRISP-Cas systems	Targeted genes	References
Golden flesh Melon	CRISPR base editors	Arg to- His mutation in the melon orange protein gene (CmOr) gene	Tzuri et al. (2015)
Tomato	CRISPR-Cas9	*STAYGREEN (SGR)* gene, a negative regulator of carotenoid biosynthesis, was knockdown	Li A. et al. (2018)
Golden Banana	CRISPR-Cas9	lycopene epsilon-cyclase (LCYε) was knockdown by creating indels	Kaur et al. (2020)
Golden rice	CRISPR-Cas9	*phytoene desaturase CrtI* and maize *Phytoene synthase* (*PSY*) genes were knocked in	Dong et al. (2020)

### Vitamin E–Enriched Crops

Vitamin E (tocopherol) is a potent lipid-soluble antioxidant and an essential component of the human diet. Many human diseases, such as cardiovascular disease and certain cancers, are associated with insufficient vitamin E intake (Rizvi et al., 2014). The daily requirement of vitamin E for humans lies between 15 and 30 mg (Ungurianu et al., 2021).

Through CRISPR-Cas9 technology, significant increase in tocopherols and tocotrienol content was achieved by targeted overexpression of Hordeum vulgare *homogentisate* phytyltransferase (*HvHPT*) and Hordeum vulgare homogentisate geranylgeranyltransferase (*HvHGGT*) (Zeng et al., 2020). These genes can be utilized for enhancing vitamin E content in other crops.

### Iron-Enriched Crops

Iron is involved in various metabolic activities such as oxygen transport and electron transport chain. Iron metabolism disorders cause the most frequent diseases in humans, encompassing multiple conditions with various clinical presentations, ranging from anemia to neurodegenerative diseases (Abbaspour et al., 2014). The iron need in humans can be fulfilled through dietary and crop biofortification (Liberal et al., 2020). CRISPR-Cas9 is responsible for the latter type of biofortification. This system has been reported to disrupt the *Inositol pentakisphosphate 2- kinase 1* (*IPK1*) gene causing iron biofortification in wheat (Ibrahim et al., 2021).

### Zn-Enriched Crops

The mineral Zn is involved in numerous cellular metabolic processes and catalytic activity of approximately 100 enzymes (Sandstead, 1994). It plays a role in immune functions (Prasad, 1995; Solomons, 1998), protein synthesis (Prasad, 1995; Heyneman, 1996), wound healing (Heyneman, 1996), DNA synthesis, and cell division (Prasad, 1995). Zinc keeps our immune system healthy (Maggini et al., 2010). Zinc maintains cell normal development and activation for innate and adaptive immune responses. It facilitates the integrity of epithelial barriers, which are essential for protecting organisms and preventing pathogen entry (Sturniolo et al., 2002; Finamore et al., 2008; Maares and Haase, 2016). Moreover, Zn can modulate the development and activity of T cells and hence be used as an immunomodulatory candidate. There is only one report on Zn enhancement in wheat, to the best of our knowledge. In this crop, the CRISPR-Cas system disrupts *Triticum aestivum Inositol Pentakisphosphate 2-kinase 1 (TaIPK1)* that reduces phytic acid to cause improvement in zinc accumulation in wheat grains (Ibrahim et al., 2021). More crops are needed to be biofortified with Zn through the CRISPR-Cas system to make available the products worldwide.

### Biofortification Through Targeting Cytokinin Metabolism

Plants absorb a range of mineral elements essential for growth, including C, H, O, N, Fe, Zn, K, Na, and others, in various forms. C, H, and O are acquired from gases or water, and their uptake pathways are straightforward and well-known (Reid and Hayes, 2003). In contrast, other elements are primarily classified as mineral elements and are mainly taken from soil in terrestrial plants or water in aquatic plants (Reid and Hayes, 2003). Root system architecture (RSA), which constitutes the structure of root length, spread, number, and length of lateral roots, is a critical developmental and agronomic characteristic that affects overall plant architecture, growth rate and yield, abiotic stress resistance, nutrient absorption, and developmental flexibility in response to environmental changes (Jung and McCouch, 2013). Phytohormones are communicators between soil and RSA, regulating root development processes, extending from organogenesis to creating higher-order lateral roots (LRs) *via* various mechanisms (Sharma et al., 2021). The hormones cytokinin (CK) and auxin (IAA), along with ethylene, are essential regulators of root growth, vascular differentiation, and root gravitropism (Aloni et al., 2006). Cytokinin negatively regulates root elongation and branching and crucially shapes RSA (Ramireddy et al., 2014). The enzymes isopentenyl transferase (IPT) present in plants regulates cytokinin levels, which are later destroyed by cytokinin oxidase/dehydrogenase (CKX) and inactivated by glucosylation through cytokinin glucosyl transferases (CGTs*)* (Chen et al., 2021). Since cytokinin level in roots negatively correlates with crop yields. The reduced cytokine level induced by the enzymes mentioned above increases root growth and uptake of mineral nutrients, particularly Zn and Fe (Chen et al., 2020). Therefore, overexpression of *CKX* and *CGTs* in the root zone increases the crop yield.

Genetically edited plants have been developed with indigenously lowered cytokinin levels that favor enrichment of P, Ca, S, Cu, Mn, Fe, and Zn in plant biomass (Ramireddy et al., 2018a). Ramireddy et al. (2018b) developed Zn-fortified field-grown barley which breaks down plant cytokinin through transgenics. In the additional study carried out by the author, the grain yield of barley was increased by knocking out CKX genes through an RNA-guided Cas9 system to generate ckx1 and ckx3 mutant lines with knockout mutations in the HvCKX1 and HvCKX3 genes, respectively. Reduced CKX activity in the ckx1 lines induced longer roots, increased surface area, and a higher number of root hairs. In contrast, enhanced CKX activity in the ckx3 mutants had opposite results. The authors’ findings show that the control of cytokinin activity is complicated, where alterations in just a single component might have unexpected consequences (Gasparis et al., 2019). In another study, silencing the OsCKX4 gene or knockout of the homologous gene OsCKX2 resulted in decreased Zn concentrations in brown rice. However, CRISPR-Cas9 mediated knockout of CYP735A involved in the formation of trans-zeatin (tZ-type) cytokinins elevates Zn concentrations (Gao et al., 2019). In yet another study, Karunarathne et al. (2022) developed the barley abnormal cytokinin response 1 repressor (HvARE1) mutants with high nitrogen content in shoots in nitrogen-deficient soil through Agrobacterium-mediated genetic transformation of immature embryos (cv. Golden Promise) with sgRNAs targeting HvARE1. Such crop types possess nitrogen use efficiency (NUE) and can reduce fertilizer input in soils, rendering them a cost-effective alternative that can prevent environmental pollution due to excessive fertilizer applications.

### Quality Improved Crops

In addition to developing nutrient-enriched crops, many crops are improved to boost production, biotic and abiotic stress resistance, and quality and nutritional value. Over several decades, innovative agricultural technology has considerably enhanced crop productivity. Consumers are more concerned about crop quality because it is linked to human health by delivering nutrients such as proteins, fiber, vitamins, minerals, and bioactive substances (Slavin and Lloyd, 2012). Compared to conventional breeding, CRISPR-based systems have increased the quality of staple, oilseed, and horticultural crops with significant accuracy and efficiency (Ku and Ha, 2020). To the best of the literature review, through CRISPR-Cas mediated genome editing, various crops have been reported for improvement in their diverse categories of quality (Table 3).

**TABLE 3:** Summary of CRISPR-Cas-based genome-edited quality improved crops.

Quality improved crops	CRISPR-Cas systems	Targeted genes	Function	References
Soybean	CRISPR-Cas9	*FAD2-1A and FAD2-1B*	Knock-down of genes *FAD2-1A* and *FAD2-1B* altered high oleic acid and low linoleic acid content	Haun et al. (2014)
Soybean	CRISPR-Cas9	*FAD2-1A and FAD2-1B*	Knock-down of genes *FAD2-1A* and *FAD2-1B* high oleic acid and low linoleic acid content	Demorest et al. (2016)
Rice	CRISPR-Cas9	*DEP1*	Negative regulator for dense erect panicles	Li et al. (2016)
Grape	CRISPR-Cas9	*IdnDH*	Knock-down of IdnDH decreased tartaric acid content	Ren et al. (2016)
Rice	CRISPR-Cas9	*GW2*, *GW5*, and *TGW6*	Negative regulator for grain length and width	Xu et al. (2016)
Rice	CRISPR-Cas9	*GW5*	Negative regulator for grain width	Liu J. et al. (2017)
Rice	CRISPR-Cas9	Starch branching enzyme (*SBEI*) and *SBEIIb*	Generation of targeted mutagenesis in *SBEI* and *SBEIIb* to create high-amylose rice	Sun et al. (2017)
Rice	CRISPR-Cas9	*OsHAK-1*	Knock down of OsHAK-1 for reduced uptake Cs^+^ from the roots	Nieves-Cordones et al. (2017)
Rice	CRISPR-Cas9	*OsNramp5*	Low Cd accumulation	Tang et al., 2017
Rice	CRISPR-Cas9	*OsNRAMP2*	Remobilization and distribution of Fe and Cd	Chang et al. (2022)
Tomato	CRISPR-Cas9	*fas, lc*	Loss-of-function mutant for regulating three major productivity traits in tomatoes; fruit size, inflorescence branching, and plant architecture	Rodríguez-Leal et al. (2017)
Tomato	CRISPR-Cas9	*ALC*	Mutagenesis and replacement in the *ALC* gene generated long-shelf-life	Yu et al. (2017)
Tomato	CRISPR-Cas9	*SlGAD2 and SlGAD3*	Deletion of the autoinhibitory domain of SlGAD2 and SlGAD3 to increase GABA content in tomatoes	Nonaka et al. (2017)
Tomato	CRISPR-Cas9	*RIN*	Knockdown of *RIN* decreased volatile organic compounds	Ito et al. (2017)
Tomato	CRISPR-Cas9	*SlAN2*	Targeted mutagenesis of *SlAN2* to understand anthocyanin biosynthesis and regulation in purple tomato cultivar “Indigo Rose”	Čermák et al. (2015), Zhi et al. (2020)
Rice	CRISPR-Cas9	Fatty acid desaturase (*OsFAD2-1*)	Targeted mutagenesis in the OsFAD2-1 gene for producing high oleic/low linoleic in rice bran oil (RBO)	Abe et al. (2018)
Rice	CRISPR-Cas9	*GS9*	The *gs9* null mutant with slender grains transformed into round grains	Zhao et al. (2018)
Wheat	CRISPR-Cas9	*α-gliadin* genes	Two sgRNAs target the *α-gliadin* gene for α-gliadin reduction, which results in low-gluten meals	Sánchez-León et al. (2018)
Wheat	CRISPR-Cas9	*TaGW2*	Negative regulator for grain size and weight	Wang et al. (2018)
Barley	CRISPR-Cas9	GBSS1 and protein targeting to Starch 1 (*PTST1*)	Generation of gbss1 and ptst1 mutants to produce starch-free endosperm	Zhong et al. (2019)
Potato	CRISPR-Cas9	steroid 16α-hydroxylase (*St16DOX*)	Knockdown of the *St16DOX* reduced the steroidal glycoalkaloids (SGA)	Nakayasu et al. (2018)
Tomato	CRISPR-Cas9	*OVATE*, *Fas*, *Fw2.2*, and *CLV3*	Edited six significant loci for yield and productivity in tomato crop lines	Zsögön et al. (2018)
Tomato	CRISPR-Cas9	*SGR1, LCY-E, Blc, LCY-B1, and LCY-B2*	Knock down of *SGR1*, *LCY-E*, *Blc*, *LCY-B1*, and *LCY-B2* increase the lycopene content	Li A. et al. (2018)
Sorghum	CRISPR-Cas9	*k1C* gene family	Knock down of genes *k1C* increased digestibility and protein quality	Li X. et al. (2018)
Carrot	CRISPR-Cas9	*F3H*	Knock down of F3H decreased anthocyanin acid content	Klimek-Chodacka et al. (2018)
Rapeseed	CRISPR-Cas9	FAD2	Knock down of *FAD2* increased oleic acid content and decreased linoleic and linolenic acid contents	Okuzaki et al. (2018)
Cassava	CRISPR-Cas9	*GBSSI*	Knock down of GBSSI decreased amylose contents	Bull et al. (2018)
Apple	CRISPR-Cas9	*IdnDH*	Mutation in *IdnDH* significantly accumulated tartaric acid	Osakabe et al. (2018)
Rice	CRISPR-Cas9	*GS3* and *Gn1a*	Negative regulator for grain size and length	Shen et al. (2018)
Lettuce	CRISPR-Cas9	*LsGGP2*	Overexpression of *LsGGP2* increased n ascorbate content	Zhang et al. (2018)
Rice	CRISPR- ABE	*GL2/OsGRF4* and *OsGRF3*	Positive regulator for grain size	Hao et al. (2019)
Wheat	CRISPR-Cas9	*TaGW7*	Negative regulator for grain width, shape, and weight	Wang D. et al. (2019)
Rice	CRISPR-Cas9	*OsGS3*, *OsGW2*, and *OsGn1a*	Negative regulator for grain size, width and weight, and number	Zhou et al. (2019)
Rice	CRISPR-Cas9	(Phospholipase D) *OsPLDα1*	Generation of mutations in the phospholipase D (OsPLDα1) gene to reduced phytic acid content as compared to their wild-type parent	Khan et al. (2019)
Maize	CRISPR-Cas9	(*Shrunken-2 gene*) *SH2*, and WX	Two sgRNAs were designed to target SH2 and WX for the generation of sweet corn and waxy corn	Dong et al. (2019)
Potato	CRISPR-Cas9 and CBE	*St*GBSS	Loss of function of the *StGBSSI* protein is related to impaired amylase biosynthesis	Veillet et al. (2019)
Sweet Potato	CRISPR-Cas9	*GBSSI*	Knockdown of *GBSSI* decreased the decreased amylase content	Wang H. et al. (2019)
Tomato	CRISPR-Cas9	*PL*, *PG2a*, and *TBG4*	Generation of mutants in the enzymes PL, PG2a, and TBG4 for increasing the shelf life	Wang W. et al. (2019)
Tomato	CRISPR-Cas9	*HYS*	Knockdown of *HYS* decreased anthocyanin content	Qiu et al. (2019)
Peanut	CRISPR-Cas9	*FAD2A* and *FAD2B*	Knock down of genes *FAD2A* and *FAD2B* increased oleic acid content	Yuan et al. (2019)
Pomegranate	CRISPR-Cas9	*PgUGT84A23* and *PgUGT84A24*	Knock down of PgUGT84A23 and PgUGT84A24 accumulated gallic acid 3-0- and 4-o-glucosides	Chang et al. (2019)
Rice	CRISPR-Cas9	*GS3* and *GL3.1*	Negative regulator for grain size and length	Yuyu et al. (2020)
Rice	CRISPR-Cas9	*OsAAP6* and *OsAAP10*	Knockout of OsAAP6 and OsAAP10 mutants were generated in japonica varieties which reduces the high grain protein content (GPC)	Wang et al. (2020)
Rice	CRISPR-Cas9	*OsGBSSI* (granule-bound starch synthase I) or waxy (Wx) gene	Downregulation of Wx expression associated with fine-tuning grain amylose content	Huang et al. (2020)
Rice	CRISPR-Cas9	*OsBADH2*	Creation of novel alleles of OsBADH2, leading to the introduction of aroma into an elite non-aromatic rice variety ASD16	Ashokkumar et al. (2020)
Rice	CRISPR-Cas9	(glutamate decarboxylase 3) *OsGAD3*	Trimming the coding region of the CaMBD domain from the *OsGAD3* gene produces higher gamma-aminobutyric acid (GABA) content	Akama et al. (2020)
Maize	CRISPR-Cas9	*waxy* gene	For fine-tuning amylase content	Gao et al. (2020)
Potato	CRISPR-Cas9	Polyphenol Oxidases (*stPPO2*)	Knockdown of the *stPPO2* reduced the browning of the tuber	González et al. (2020)
Tomato	CBE	*SlDDB1, SlDET1, SlCYC-B*	Upregulation of *SlDDB1*, *SlDET1*, *and SlCYC-B* increased carotenoid, lycopene, and β carotene	Hunziker et al. (2020)
Tomato	CRISPR-Cas9	*SlANT2, SlAN2-like*	Knockdown of *SlANT2*, *SlAN2-like* decreased anthocyanin content	Zhi et al., 2020; Yan et al., 2020
Tomato	CRISPR-Cas9	*FLORAL4*	Mutagenesis of *FLORAL4* increased phenylalanine-derived volatile content	Tikunov et al. (2020)
Tomato	CRISPR/LbCpf1	Salt-tolerant SlHKT1; 2 allele	Homology-directed repair (HDR)–based genome editing for salt tolerance	Vu et al. (2020)
Tomato	CRISPR-Cas9	*EXCESSIVE NUMBER OF FLORAL ORGANS* (*ENO*)	The *ENO* gene mutation gives rise to plants that yield larger multilocular fruits due to an increased size of the floral meristem	Yuste-Lisbona et al. (2020)
Soybean	CRISPR-Cas9	*Gly mBd 28 K* and *Glym Bd30 K*	Knock down of *genes GlymBd 28 K* and *GlymBd 30 K* produces hypoallergenic soybean plants	Sugano et al. (2020)
Eggplant	CRISPR-Cas9	*PP04*, *PPOS*, and *PP06*	Knock down of *PP04*, *PPOS*, and *PP06* decreased browning	Maioli et al. (2020)
*Brassica rapa*	CRISPR-Cas9	*BrOG1A and BrOG1B*	Knock down of *BrOG1A and BrOG1B* decreased fructose and glucose and increased sucrose contents	Jiang et al. (2020)
Rapeseed	CRISPR-Cas9	*SFAR4* and *SEARS*	Knock down of SFAR4 and SEARS increased oleic acid content and decreased linoleic and linolenic acid contents	Karunarathna et al. (2020)
Rapeseed	CRISPR-Cas9	*BnTT8*	Knock down of *BnTT8* altered the oil content and fatty acid composition	Zhai et al. (2020)
Rapeseed	CRISPR-Cas9	*BnITPK*	Knock down of *BnITPK* decreased phytic acid content	Sashidhar et al. (2020)
Tomato	CRISPR-Cas9	*SlAN2*	Targeted mutagenesis of *SlAN2* to understand anthocyanin biosynthesis and regulation in purple tomato cultivar “Indigo Rose”	Zhi et al. (2020)
Rice	CRISPR-Cas9	*GW2*	Negative regulator for grain length and width	Achary and Reddy, (2021)
Rice	CRISPR-Cas9	*OsSPL16/qGW8*	Negative regulator for the grain size	Usman et al. (2021)
Maize	CRISPR-Cas9	*CLE genes*	Engineered *CLE* genes are used to increase the yield	Liu L. et al. (2021)
Maize	CRISPR-Cas9	*ZmBADH2a* and *ZmBADH2b*	Generation of *zmbadh2a* and *zmbadh2b* single mutants and the *zmbadh2a-zmbadh2b* double mutant for popcorn-like scent in seeds from the double mutant, but not from either single mutant or in wild type	Wang Y. et al. (2021)
Potato	CRISPR-Cas9	sterol side chain reductase 2 (*StSSR*)	Successfully edited the *StSSR2* gene of tetraploid cultivated potatoes to reduce toxic SGA.	Zheng Z. et al. (2021)
Potato	CRISPR-Cas9	Starch branching enzyme (*Sbe*)	Generation of mutation in one or two *Sbe* genes to develop an increased amylose ratio and long amylopectin chains	Zhao et al. (2021)
Tomato	CRISPR-Cas9	*SlINVINH1* and *SlVPE5*	Knock down of genes (*SlINVINH1* and *SlVPE5*) increased glucose, fructose, and total soluble solids (TSS)	Wang B. et al. (2021)
Camelina	CRISPR-Cas9	CsFAD2	Knock down of CsFAD increased oleic acid contents	Lee et al. (2021)
Banana	CRISPR-Cas9	*MaACO1*	Knock down of *MaACO1* increased shelf life	Hu et al. (2021)
Rice	CRISPR-Cas9	*OsNRAMP2*	Remobilization and distribution of Fe and Cd	Chang et al. (2022)
Rice	CRISPR-Cas9	(betaine aldehyde dehydrogenase 2) *OsBADH2*	Loss of function (*OsBADH2*) affects the biosynthesis of 2-acetyl-1-pyrroline (2-AP), which is responsible for the aroma in fragrant rice	Hui et al. (2022)

## Conclusion and Future Prospects

The CRISPR-Cas system is an efficient, convenient, and cost-effective genome editing tool through which major crops can be biofortified for deficient vitamins or minerals. Furthermore, due to the non-insertion of foreign DNA and lesser regulatory restrictions, the products of this technology are easily acceptable by people in society. This genome editing tool has immense potential to eliminate the nutrient deficiency of crops and provide food security for the ever-increasing population. So far, CRISPR technology has been specifically utilized to modify a single gene for crop improvement. However, it holds the potential to manipulate several genes simultaneously, either by assembling multiple sgRNA expression cassettes in a single CRISPR vector or by producing more RNA through an endogenous RNA processing system.

There are several challenges to the widespread CRISPR-based agriculture revolution which include varying legislation and regulatory frameworks for gene-edited crops, delivery of CRISPR-Cas payloads, and off-target activity in CRISPR-Cas systems. In this direction, the United States, Argentina, Brazil, Chile, and Colombia have established product-based regulations by illustrating that gene-edited crops are free from GMO monitoring, provided the end products contain no exogenous DNA. Furthermore, 13 World Trade Organization countries have issued a declaration favoring gene editing for agricultural innovation, marking the first step toward drafting a worldwide regulatory framework.

Recently in India, the Department of Biotechnology, Ministry of Science and Technology has come up with “*Guidelines for Safety Assessment of Genome Edited Plants, 2022*” (https://ibkp.dbtindia.gov.in/Page Content/ShowBrowsedFile? FileName=20220521202 445079_Final11052022Annexure%20I,%20Genome_Edited_Plants_2022_Hyperlink.pdf&FPath=E:\\DBT_Content_Test\CMS\Guidelines/20220521202445079_Final11052022Annexure%20I,%20Genome_Edited_Plants_2022_Hyperlink.pdf), which provides a road map for the development and sustainable use of genome editing technologies in India, specifying the biosafety and/or environmental safety concerns and describing the regulatory pathways to be adopted while undertaking the genome editing of plants.

Another limitation of CRISPR technology is off-target changes in the host genome, which is needed to be rectified through alteration in the current CRISPR-Cas methodology to minimize off-target binding for optimizing sgRNA, Cas proteins, and delivery methods. Inclusion of highly specific sgRNAs may lower off-target rates. Other strategies, including the extension of the sgRNA sequence and 3′-terminal cleavage of the sgRNA, may reduce the off-targeting effect^1^.

Despite the persisting challenges, several essential crops have been altered using CRISPR-Cas for nutrition, quality, and productivity enhancement. Nonetheless, additional study is needed to investigate more crop diversity in terms of nutrition, quality, and productivity enrichment to identify effective biofortification targets and optimize CRISPR delivery methods. The CRISPR-based agricultural genome editing is the future of crop fortification as it can design genes for increased vitamin synthesis, crop quality features, and crop production qualities. CRISPR-based genome editing has a great potential to achieve the 2030 goal of eradicating hunger, food insecurity, and all forms of human malnutrition.

## Footnotes

1
https://www.who.int/news-room/fact-sheets/detail/malnutrition
